# NUMBER AND REACH OF INTERVENTIONS IN BEST PROGRAMS FOR CAREGIVING

**DOI:** 10.1093/geroni/igae098.0036

**Published:** 2024-12-31

**Authors:** David Bass, Zoe Fete, Megan Huth, Rachel Cannon, Morgan Minyo, Sara Powers

**Affiliations:** Benjamin Rose, Cleveland, Ohio, United States; Benjamin Rose, Cleveland, Ohio, United States; Benjamin Rose, Cleveland, Ohio, United States; Benjamin Rose, Cleveland, Ohio, United States; Benjamin Rose Institute on Aging, Cleveland, Ohio, United States; Benjamin Rose, Cleveland, Ohio, United States

## Abstract

A major advance in Gerontology is the development of numerous evidence-based support programs for dementia family and friend caregivers. However, a common untested assumption is that these proven programs are not widely available to caregivers. Data collected for the new version of Best Programs for Caregiving (BPC) for family and friend caregivers afforded an opportunity to carefully document the scaling and reach of the 47 evidence-based programs in BPC. The first phase of this research surveyed program-developers to identify all healthcare and community organizations delivering their programs in 2022/2023. 600 service organizations were identified as offering a BPC program (nearly twice the number identified in 2019). The second phase of research surveyed the 600 delivery organizations to verify that programs were being delivered and to collect related data, such as geographic areas served. 252 (42%) of the 600 organizations were verified as delivering a BPC program. The geographic areas served ranged from a single city or county to the entire U.S. Notably, 25 organizations served caregivers nationally via remote program delivery. Reasons the 348 (58%) delivery organizations that were identified by developers, but not verified as delivering programs, included: stopped the program because of funding or staffing challenges, never delivered the program, or could not be reached. Results suggest more programs are available and have a wider reach to caregivers than expected, but the distribution of programs could be improved, if developers had more capacity or assistance with marketing, tracking, and monitoring program implementations.

